# An Integrated, Exchangeable Three-Electrode Electrochemical Setup for AFM-Based Scanning Electrochemical Microscopy

**DOI:** 10.3390/s23115228

**Published:** 2023-05-31

**Authors:** Andreas Karg, Sebastian Gödrich, Philipp Dennstedt, Nicolas Helfricht, Markus Retsch, Georg Papastavrou

**Affiliations:** 1 Physical Chemistry II, University of Bayreuth, 95447 Bayreuth, Germany; 2 Bavarian Institute for Battery Technology, University of Bayreuth, 95448 Bayreuth, Germany; 3 Physical Chemistry I, University of Bayreuth, 95447 Bayreuth, Germany

**Keywords:** scanning electrochemical microscopy, atomic force microscopy, electrochemical sensors

## Abstract

Scanning electrochemical microscopy (SECM) is a versatile scanning probe technique that allows monitoring of a plethora of electrochemical reactions on a highly resolved local scale. SECM in combination with atomic force microscopy (AFM) is particularly well suited to acquire electrochemical data correlated to sample topography, elasticity, and adhesion, respectively. The resolution achievable in SECM depends critically on the properties of the probe acting as an electrochemical sensor, i.e., the working electrode, which is scanned over the sample. Hence, the development of SECM probes received much attention in recent years. However, for the operation and performance of SECM, the fluid cell and the three-electrode setup are also of paramount importance. These two aspects received much less attention so far. Here, we present a novel approach to the universal implementation of a three-electrode setup for SECM in practically any fluid cell. The integration of all three electrodes (working, counter, and reference) near the cantilever provides many advantages, such as the usage of conventional AFM fluid cells also for SECM or enables the measurement in liquid drops. Moreover, the other electrodes become easily exchangeable as they are combined with the cantilever substrate. Thereby, the handling is improved significantly. We demonstrated that high-resolution SECM, i.e., resolving features smaller than 250 nm in the electrochemical signal, could be achieved with the new setup and that the electrochemical performance was equivalent to the one obtained with macroscopic electrodes.

## 1. Introduction

Scanning electrochemical microscopy (SECM) is a well-established analytical technique that allows measuring electrochemical sample properties on the micro- and nanometer-scale [1,2,3,4,5,6]. SECM belongs to the so-called scanning probe techniques and the basic idea of SECM is to scan a small electrode over a sample and to detect in a locally resolved manner changes in current as a function of the lateral position on the sample and the distance to the sample, respectively. The technique has been presented first in 1989 [7]. In the following decades, many different imaging modes have been developed [1,3]. However, independent from the imaging mode, some fundamental instrumental parameters determine the performance in SECM measurements. The parameters compromise the probe, the sample topography, the distance control between probe and sample, and the possibility to track and follow the surface topography of the sample [3]. Tracking surface topography and thus surface separation distance is essential as these have a strong influence on the electrochemical currents that are the primary measurement signal [4].

Originally, SECM was based solely on micropipettes, which were scanned over the sample [7,8,9,10]. However, micropipette-based SECM was strongly limited in determining the surface topography in a non-destructive manner and with high lateral resolution [11,12]. In particular, it was and is difficult to control the force exerted on the sample during the imaging process, which is carried out mostly by shear-force detection [9]. The method of micropipette-based SECM is still under active development and some limitations have been overcome [13,14]. SECM probes from micropipettes provide also a number of advantages in terms of versatility of the probes and tuning them from specific applications, such as the detection of local ions. Hence, micropipettes in SECM have found applications in studying corrosion [15], catalysis [16], biological cells, or battery materials [17,18,19]. In particular, in the field of scanning ion conductance microscopy micropipette-based techniques are extensively used [14].

The advent of AFM-based SECM allowed us to overcome many problems associated with micropipettes, resulting in a high lateral resolution as well as a non-destructive imaging. Moreover, the simultaneous acquisition of laterally resolved data on additional sample properties, such as adhesion or elastic response, is a further advantage of AFM-based SECM approaches. Unfortunately, AFM-SECM has long suffered from the difficult and tedious preparation of probes that combine a sharp AFM tip and an electrode on one cantilever [20,21,22,23,24,25,26]. Recently, batch preparation techniques for the fabrication of such SECM-cantilevers have been improved significantly and suitable cantilevers became available more widely [12,25,26,27]. In consequence, AFM-based SECM has been recently utilized increasingly in several fields such as battery [17,18] and corrosion research, catalysis [28], and biology [5].

Electrodes represent a crucial element not only in SECM but in all electrochemical experiments and electrode arrangements electrochemical cells are often of great importance [29,30,31]. In the case of SECM, not only the electrochemical sensitivity but also the spatial resolution in the electrochemical signal depends critically on the dimension and geometry of the working electrode, which is scanned as a probe over the sample surface. Therefore, it is not surprising, that the fundamental combined AFM-SECM sensors have recently received much attention [32]. The outline of the entire electrochemical cell is also of great importance for the performance of the instrumental setup [29]. The design of electrochemical cells for scanning probe microscopy has received only moderate interest in the past [33,34]. On the other hand, by now, electrochemical cells are commercially available from most AFM manufacturers. However, common to all standard designs for all AFM electrochemical cells is that they concentrate in their design on macroscopic working electrodes that are often used as samples. Hence, the position and the dimensions of the reference and counter electrode in the electrochemical cell are designed with respect to the second macroscopic working electrode. 

However, the presence of macroscopic electrodes in the fluid cell are not necessary or even advantageous for all applications of SECM [35]. For various applications, the SECM-probe is the only or the primary working electrode, i.e., there is no need to keep a macroscopic sample of several mm^2^-area under potentiostatic control. In this case, the requirements with respect to the area of the counter electrode and the position of the reference electrode change substantially as the AFM-SECM probe corresponds to a nanoelectrode, which is incorporated into the AFM-cantilever. There are two general important rules in relation to electrochemical cells. First, the size of the counter electrode should be several times larger than the one of the working electrodes. Secondly, the reference electrode should be placed as close as possible to the working electrode, which is a requirement that leads to the use of devices like the Luggin-capillary [29]. Adapting these requirements to the nm-sized electrode on the AFM-cantilever leads to drastically new boundary conditions in designing the counter and reference electrodes, respectively, and their positions in the electrochemical cell. The counter electrode can be reduced significantly in size, and both the counter and the reference electrode can be placed on the glass packaging of the AFM-cantilever. This approach, which we refer to as *SECM-Cantilevers/Echemcell,* in which the cantilever and cell are now one unit, is based on pastes for screen-printed electrodes [36]. The latter became increasingly popular for analytical applications and provide reliable as well as inert electrode materials [37,38,39,40,41,42,43,44,45]. This new type of electrochemical cell for AFM-based SECM has been characterized for its electrochemical performance. We performed two types of experiments for this characterization. On one hand, cyclic voltammetric measurements with a well-defined redox couple allow us to verify not only the electrode on the SECM cantilever but also the function of the other electrodes by comparison of the cyclic voltammograms. By detecting the currents on the working electrode, e.g., the SECM-tip, as a function of the applied potential and comparing these currents to the ones reported previously for the same redox-couple in the liquid phase, the electrochemical properties of the electrode as well the other electrodes can be verified in an exemplary manner. On the other hand, SECM imaging with the newly developed cell and the comparison to the images as acquired with a standard cell provides additional verification for the suitability of the new cell in SECM-imaging applications. 

## 2. Materials and Methods

### 2.1. Preparation of Integrated SECM-Cantilevers/Echemcell

Commercial SECM-AFM cantilevers (Bruker, Santa Barbara, CA, USA) were painted by means of a Pt-paste (Platinum Polymer Paste, Sun Chemical Corp., Parsippany-Troy Hills, NJ, USA) and an Ag/AgCl-paste (60% Ag/40% AgCl in paintable format, Zimmer & Peacock, Royston, UK) through a homemade rubber-mask, resulting in an electrode geometry of approx. length 7 mm and approx. width 0.6 mm, respectively. Afterwards, the modified cantilever was dried for 30 min at 80 °C. The painted electrodes were electrically contacted using a PEI-insulated silver wire (0.125 mm in diameter, Advent Research Materials Ltd., Oxford, England) using conductive silver (Acheson 1415, Plano GmbH, Wetzlar, Germany) and UV-curing glue (NOA 63, Norland Products Inc., Jamesburg, NJ, USA). An additional insulating layer (Red insulating Varnish GC Waldom, GC Electronics, Rockford, IL, USA) was applied to the contacts. Scanning electron images were taken by means of a LEO1530 SEM (Carl Zeiss Microscopy GmbH, Oberkochen, Germany). Prior to the SEM measurements, the SECM cantilevers were sputtered with a layer of approx. 10 nm of carbon. 

### 2.2. Electrochemical Characterization

Cyclic voltammetric measurements were performed by applying a potential via a CHI750i (CH Instruments, Inc., Austin, TX, USA). For long-term stability measurements, a circular Pt-macroelectrode (1 mm in diameter, Metrohm AG, Herisau, Switzerland) was used as a working electrode. A glass slide that has been cleaned in an ultrasonic bath in aqueous 2vol% Hellmanex solution (Hellmanex III, Hellma GmbH & Co. KG, Müllheim, Germany) was painted with Pt-paste and Ag/AgCl-paste in an analogous manner compared to the integrated SECM-cantilevers/Echemcell. These electrodes were applied as counter electrodes and reference electrodes, respectively. Cyclic voltammograms were recorded in an aqueous solution of 5 mM potassium ferrocyanide (99.95%, Sigma-Aldrich Inc., St. Louis, MO, USA), 5 mM potassium ferricyanide (99.98%, Sigma-Aldrich Inc, St. Louis, MO, USA) and 0.1 mM KNO_3_ (99%, abcr GmbH, Karlsruhe, Germany) every 30 min for 3 h at a scan rate of 0.05 V/s. The experiments have been carried out under standard experimental conditions (room temperature and atmospheric pressure).

For the comparison of the electrochemical properties, also the integrated SECM-cantilever/Echemcell was characterized by cyclic voltammetry, where the electroactive cantilever tip was used as working electrode. In an electrochemical AFM cell, cyclic voltammetric measurements in an aqueous solution of 5 mM hexaammineruthenium(III) chloride (99%, abcr GmbH, Karlsruhe, Germany) and 0.1 mM KNO_3_ (99%, abcr GmbH, Karlsruhe, Germany) were performed at a scan rate of 0.02 V/s with the painted electrodes connected as counter and reference electrodes to test the integrated SECM-cantilever/Echemcell. For comparison experiments, a chlorinated silver wire as a quasi reference electrode (0.125 mm in diameter, Advent Research Materials Ltd., Oxford, England) and a coiled Pt-wire (0.127 mm in diameter, Advent Research Materials Ltd., Oxford, England) were used as a counter electrode to compare it to a standard SECM setup. The wire electrodes and painted electrodes were referenced against a commercial saturated calomel electrode (RE-2B, Basi Inc., West Lafayette, IN, USA) using a high-resistance voltmeter (Keithley Instruments, Solon, OH, USA).

### 2.3. Preparation of Gold Nano-Meshes

Monolayers of spherical polystyrene beads (1.04 ± 0.04 μm in diameter, Microparticles GmbH, Berlin, Germany) were produced as described by Retsch et al. [46]. Glass slides were cleaned for 15 min in an ultrasonic bath with a 2vol% aqueous of Hellmanex III (Hellma GmbH & Co. KG, Müllheim, Germany), extensively rinsed with ultrapure water and dried under a nitrogen stream. The glass slides were functionalized by means of a liquid phase silanization for 1 h with an aqueous 1 vol% of a silane (N-trimethoxysilylpropyl-N,N,N-trimethylammonium chloride, 50% in methanol, abcr GmbH, Karlsruhe, Germany). Cationically functionalized glass slides were spin-cast with a 3 wt% polystyrene particle dispersion at a rotation speed of 4000 rpm. Freely floating monolayers were assembled at the air/water interface by slow immersion of the particle-coated glass substrate into a 0.1 mM SDS solution in MilliQ water. The aqueous phase was adjusted to pH 12 by adding a few drops of ammonia. The floating monolayer was transferred to a glass substrate and dried in air. Based on the approach of Stelling and coworkers, nanomeshes were produced from these monolayers [47]. The prepared monolayers were etched in a plasma reactor MiniFlecto (Plasma Technology GmbH, Herrenberg-Gültstein, Germany) with 75% argon and 25% oxygen at 80 W power at a pressure of 0.14 mbar. Etching was conducted for 20 min in order to obtain non-close packed monolayers with particles of 870 nm in diameter. A thin layer of chromium (thickness = 3 nm) as an adhesion promoter and a consecutive layer of gold (thickness = 50 nm) were deposited by thermal evaporation using a Balzers BA360 (Oerlikon Balzers Ltd., Balzers, Liechtenstein). The layer thickness was monitored via a SQM 160 microbalance (Sigma Instruments, INFICON Holding AG, Bad Ragaz, Switzerland). The remaining particles were removed using Scotch^®^ tape (3M corp-. Saint Paul, MN; USA) leading to nanohole arrays in the gold film. These Au-nano-mesh substrates were cleaned for 10 min in an ultrasonic bath with a 2% aqueous Hellmanex III (Hellma GmbH & Co. KG, Müllheim, Germany) solution in ultrapure water. The surfactant was extensively rinsed off with ultrapure water and the substrates were placed in ethanol in an ultrasonic bath for 10 min and dried under a nitrogen stream.

### 2.4. SECM Measurements

Height and current images of the AFM-SECM measurements were acquired with a Dimension ICON (Bruker Corp., Billerica, MA, USA) equipped with a Nanoscope V controller (Bruker Bruker Corp., Billerica, MA, USA) in a partially closed electrochemistry fluid cell. This cell has been purposely designed on base of a commercially available electrochemical cell (Asylum Research Corp., Santa Barbara, CA, USA). The feed-throughs for the liquid exchange have been used for connection to the electrodes inside the cell. A homemade reference electrode from an Ag/AgCl-wire has been prepared by the following procedure. From PTFE-insulated Ag wires with a diameter of 0.125 mm (Advent Research Materials Ltd., Oxford, England) the insulation has been partially removed and the resulting free area has been electrochemically coated with a layer of AgCl using an automatic chlorination device (AC1-01, npi electronic GmbH, Tamm, Germany). As a counter electrode, a coiled Pt-wire (0.127 mm in diameter, Advent Research Materials Ltd., Oxford, England) with a total effective length of about 100 mm has been used. 

When AFM-SECM measurements with integrated SECM-cantilevers/Echemcells were performed, a painted Ag/AgCl electrode acted as reference and a painted Pt-electrode acted as counter-electrode, respectively. The electrochemical-active SECM-cantilevers were used as primary working electrodes, while the gold-nanomesh substrates were used as secondary working electrodes, respectively. A constant potential of −0.5 V was applied to the SECM-tip, while a potential of 0.2 V was applied to the gold-nanomesh for regeneration of the reduced species by means of a CHI750i potentiostat. The AFM-SECM measurements were performed in an aqueous solution of 5 mM hexaamminerruthenium(III) chloride with 0.1 M KNO_3_ in PeakForce lift mode at a lift height of 40 nm. In order to eliminate line noise in the current signal, an active compensation device (HumBug noise eliminator, Quest Scientific Instruments Inc., North Vancouver, BC, Canada) was used. AFM height and current images were processed with NanoScope Analysis 1.80 (Bruker Bruker Corp., Billerica, MA, USA). The resulting current images were normalized to the current on the gold line-by-line, which was evaluated by means of a home-built procedure in IgorPro (Wavemetrix, Inc., Lake Oswego, OR, USA).

## 3. Results

### 3.1. An Electrochemical Cell Integrated into the Glass Packaging of an AFM-SECM-Cantilever

**Figure 1:**
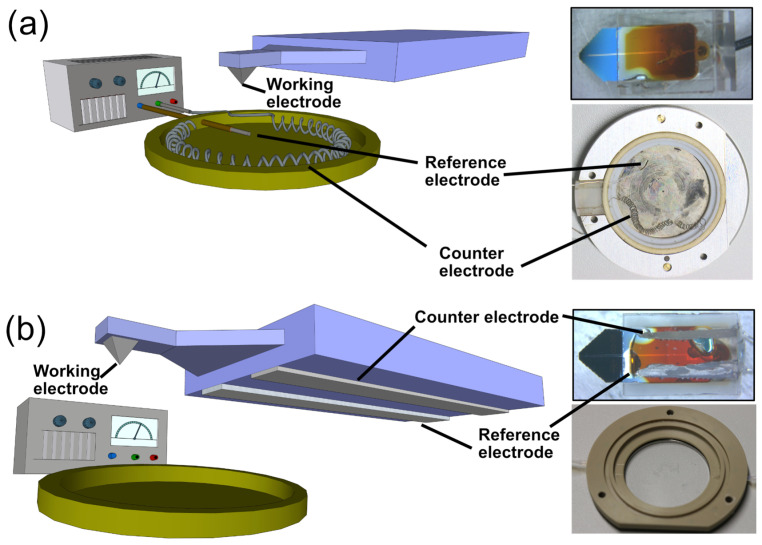
(**a**) Left: Schematic representation of the ‘classical’ setup for AFM-based SECM with macroscopic electrodes. A commercial AFM-SECM cantilever is connected to a potentiostat as one working electrode, while the sample is the second working electrode. The electrochemical cell is based on Ag/AgCl- and Pt-wires as quasi-reference and counter electrodes, respectively. Right: photographs show the commercial SECM-cantilever, as well as the electrochemical cell with reference and counter electrode. (**b**) Left: Schematic representation of the integrated SECM cantilever/Echemcell setup based on the commercial AFM-SECM cantilever and screen-printed electrodes on its glass packaging. Here, the working, reference and counter electrode are fully integrated into the SECM-cantilever chip (right, top). For a three-electrode experiment, no additional wiring in the fluid is required. Hence, a standard fluid cell is viable for measurements (right, bottom).

In AFM-SECM feedback mode, the SECM-cantilever is used for imaging as well as for characterizing the electrochemical properties of a sample surface [7]. Here, we present a newly developed electrochemical cell in direct integration with the SECM-cantilevers and their glass packaging. More specifically, we integrated the reference and the counter electrode directly on the cantilever glass packaging. Therefore, both were brought in the direct vicinity of the working electrode used for SECM imaging. More details will be given below. In order to test these integrated SECM-cantilevers/Echemcells, we compared them in their performance to ‘classical’ electrochemical cells. A ‘classical’ electrochemical cell is shown in a schematic representation in Figure 1a. Similar electrochemical cells for applications in AFM have been used in our groups for many years [48,49,50,51]. The design is based on the standard distribution of electrodes for electrochemical cells in AFM [20,23]. The large sample with dimensions of ca. 10 mm × 10 mm, which can act also as a working electrode, is located at the center of the cell. The corresponding counter electrode is placed in an approximately semi-circular configuration around the sample. We commonly utilize either Pt-wires, which we form in a spiral shape to increase the surface area, or Pt-nets as counter electrodes. The former has been utilized in the experiments presented here. As counter electrodes commonly either commercial solid-state reference electrodes (e.g., DriRef^®^) or silver wires that have been chlorinated, have been used in combined AFM/electrochemistry setups [20,23,52].

For this study, the SECM-cantilever, namely the electrode acting also as an AFM tip, acts as the primary working electrode. For some experiments, the large macroscopic electrode is used as 2nd working electrode. Electrochemical control of one or two working electrodes has been provided by means of a commercial bi-potentiostat. An image of the complete, unmodified SECM-cantilever, including the packaging made from glass, is shown in Figure 1a top right. This glass carrier is also used to immobilize the cantilever in the cantilever holder of the AFM head. In the electrochemical cell for the AFM (cf. Figure 1a), the counter electrode was a platinum wire, which has been coiled in order to increase the available surface area (cf. Figure 1a right bottom). A chlorinated silver wire (Ag/AgCl) serves as reference electrode [12,48,49]. For measurements at low ionic strength, the wire allows for a rather symmetric distance around the macroscopic working electrode located in the center of the fluid cell (cf. inset Figure 1a right bottom). The type of components shown in the images of Figure 1a represents the classical setup for AFM-based SECM and has been utilized in several studies from us [12] or other groups [53,54].

Figure 1b shows the newly developed integrated SECM-cantilever/Echemcell, which places all three electrodes on the cantilever and its glass packaging, respectively. The schematic illustration (cf. Figure 1b left) shows the position of these electrodes. Under the condition that only the nm-sized electrode on the AFM-tip has to be electrochemically controlled, a more miniaturized setup becomes feasible, and the counter and reference electrode can be much smaller. Moreover, both electrodes can be placed much closer in the vicinity of the working electrode at the AFM-SECM tip. The inset on the right top of Figure 1b shows the same type of SECM-cantilevers as in Figure 1a (inset, top right) but this time includes the two additional electrodes, namely counter and reference electrode, that have been painted on the glass packaging. These on-substrate electrodes eliminate the need for additional electrodes in the measurement cell. Therefore, measurements in standard fluid cells (cf. Figure 1b right bottom) or even petri-dishes can be conducted. The detailed preparation and characterization of the integrated SECM-cantilevers/Echemcells will be given in the following.

### 3.2. Preparation of AFM-SECM Cantilevers with Integrated Electrochemical Cell

**Figure 2:**
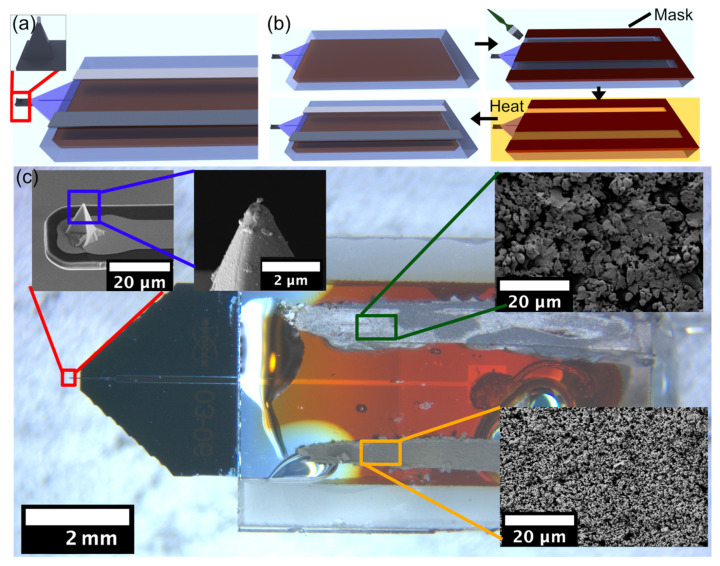
(**a**) Schematic representation of the integrated SECM-cantilever/Echemcell developed in this study. Inset: Ultramicroelectrode on the tip used for both imaging and electrochemical characterization. (**b**) Schematic representation of the preparation steps. (**c**) Optical microscopy image at low magnification of the integrated SECM-cantilever/Echemcell. The insets show scanning electron microscopy images of the combined AFM-SECM tip, as well as the microstructures of the dried Ag/AgCl-paste (dark green, top) and dried Pt-paste (orange, bottom).

Figure 2 summarizes the preparation of a three-electrode electrochemical cell, which is integrated into commercial AFM-SECM cantilevers and their glass packaging. In Figure 2a, the general setup is shown in a schematic overview. The combined AFM-SECM cantilever has an isolated Pt-tip at its apex. This tip acts as a working electrode and is already present in the commercially available cantilever [12]. The two additional electrodes required for a complete electrochemical cell are situated on the glass packaging and must be added afterwards. Figure 2b illustrates the necessary preparation process for these electrodes on the glass substrate. Starting from the bare glass surface (cf. Figure 2b left top), a custom-made rubber mask is placed on the substrate. The mask from high-purity silicon rubber has openings with the outline of the two electrodes. In the following, the electrodes were painted on the substrate. The paints for the electrodes have been originally developed for screening printing processes but can also be deposited with a fine brush. For the counter electrode, we used a Pt-based paste and for the reference electrode an Ag/AgCl-based paste. Further details are given in the experimental section. The deposited pastes have been cured at high temperature (80 °C) followed by removal of the mask (cf. Figure 2b bottom). Finally, the two additional electrodes have been contacted by thin insulated wires. We used a procedure for the wire attachment and insulation that has been developed to produce electrochemical colloidal probes (cf. experimental section) [51].

Figure 2c shows the resulting integrated SECM-cantilever/Echemcell and its glass packaging. The low magnification overview image serves as orientation (Figure 2c central). Scanning electron microscopy images of the working electrode on the apex of the tip are shown in Figure 2c left top and center. Please notice that the integrated SECM-cantilever/Echemcell has been utilized for measurements and that the debris visible results from the transfer through the water/air interface and drying. Further SEM images show the microstructures of the dried Ag/AgCl paste (cf. Figure 2c, top right) and the Pt-paste (cf. Figure 2c, bottom right), which are used as a reference and counter electrode, respectively. For both, grains with defined borders are visible, while the grain size is significantly smaller for the Pt-paste. The configuration of the electrodes resembles slightly the one encountered for in situ electrochemical cells for transmission electron microscopy [30,55,56].

### 3.3. Characterization by Cyclic Voltammetry

In order to evaluate the electrochemical performance of the integrated SECM-cantilever/Echemcell we did perform a number of cyclic voltammetry measurements. Cyclic voltammetry (CV) is a standard analytical technique that allows us to follow the oxidation and reduction of electroactive species at an electrode under potentiostatic control while applying a time-dependent triangular voltage signal and acquiring the resulting current. The CV technique is routinely used to characterize (ultra-)microelectrodes utilized for SECM probes [23,54,57] by conducting CV measurements with standard redox couples, such as hexacyanoferrate or ruthenium complexes, which have been extensively described in the literature [29,57]. In particular, CV is also useful to verify the function of nanoelectrodes, such as the electrodes on SECM cantilevers [57,58]. In consequence, CV allows also to verify the experimental setup, i.e., the entire electrochemical cell and not only the state of the nanoelectrode [59]. It should be pointed out that the following CV experiments are not intended to provide a statistical analysis, as the electrochemical process is already well-described [57]. This approach of performing measurements for a selected redox-couple is standard for the development of novel SECM probes [57]. By demonstrating that previously reported CVs can be reproduced the newly developed setup is benchmarked as any significant change in the electrochemical behaviour of the electrodes would lead in this respect to significant changes. In particular, this approach allows us to address also the stability of the electrode materials [57].

**Figure 3:**
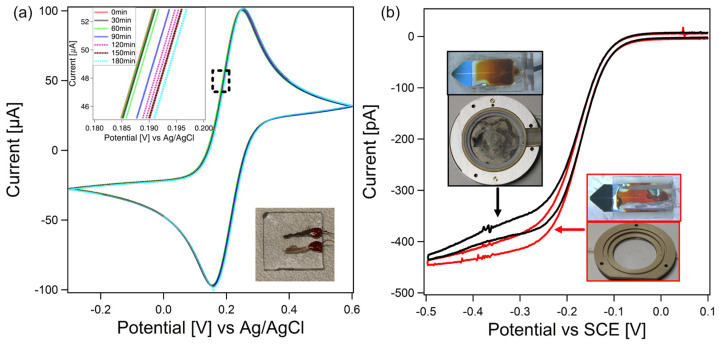
(**a**) Long-term stability experiment, using a commercial Pt-macroelectrode as working electrode and Ag/AgCl- and Pt-electrodes prepared analogously to the integrated SECM-cantilever/Echemcell. Cyclic voltammograms in ferricyanide (5 mM)/ferrocyanide (5 mM) were acquired every 30 min. On the macroscopic scale, no shift in the CVs could be detected. Inset: Zoom-in into long term CV, showing a difference of the CVs smaller than 6 mV over 3 h. No direct correlation between shift and time could be traced. (**b**) CVs of the AFM-SECM setup in hexaammineruthenium(III) chloride, a standard electrolyte used in AFM-SECM measurements [12,54], using the cantilever tip as ultramicroelectrode. Black data: CVs as acquired by means of a ‘classical’ electrochemical fluid cell for AFM using Ag/AgCl- and Pt wires as reference and counter electrodes. Red data: CVs acquired solely by integrated SECM-cantilever/Echemcell.

In the first set of experiments, the electrochemical responses of the electrodes prepared by the here-presented pastes have been verified. The paste-based electrodes have been attributed the same roles as the integrated SECM-cantilever/Echemcell. The counter electrode has been prepared by the Pt-paste and the reference electrode by the Ag/AgCl-paste. The working electrode was a commercial circular Pt-macroelectrode with an electrode area of approximately 0.8 mm^2^. This Pt-macroelectrode was located in close vicinity to a glass substrate on which the Pt- and Ag/AgCl-paste were prepared in a completely analogous manner to the glass packaging of the SECM cantilevers. An image of the electrode setup is shown as an inset in Figure 3a (cf. for comparison to Figure 2 central). A cyclic voltammetric measurement with standard parameters (cf. Materials and methods) was repeated every thirty minutes for a total time interval of 3 h while keeping the electrode configuration constant during the whole time. The corresponding CVs in Figure 3a show the overall shape as expected for a macroelectrode with pronounced oxidation and reduction peaks. The position of the peaks and current densities are completely in line with the ones reported in literature for the used ferro/ferricyanide redox couple [60,61]. Moreover, Figure 3a demonstrates the long-term stability of the paste-based electrodes (cf. inset on the left side of the cyclic voltammogram). The absence of any further peaks in the cyclic voltammogram, indicating the absence of contaminations in the paste electrodes, should be noted as well.

Figure 3b shows a second set of CV experiments where the electrochemical performance of the electrodes of the integrated SECM-cantilever/Echemcell have been compared to the ones of a conventional setup using macroelectrodes. In this case, the working electrode was the SECM tip. Another CV with hexaammineruthenium(III) chloride has been performed. The different shape of the cyclovoltammogram in Figure 3b compared to Figure 3a has its origins in the very different dimensions of the working electrode. The sigmoidal shape is commonly observed for nanoelectrodes [57,58]. The CV in Figure 3b corresponds to the ones reported previously, which have been acquired with a similar type of SECM-cantilevers and the same redox couple [12,54]. Practically, no difference is observed between the two sets of experiments, one performed with standard setup (SECM-cantilever, macroscopic reference and working electrode in cell, cf. Figure 3b inset top, black curve) and the here-developed setup (integrated SECM-cantilever/Echemcell only, cf. Figure 3b inset bottom, red curve). We could not observe any offset in terms of the CV as acquired by the two electrochemical setups while the working electrode, i.e., the SECM-cantilever, remained constant. Moreover, no difference in peak height and shape in the two different CVs could be detected. From these results for an exemplary electrochemically active substance (i.e., hexaammineruthenium(III) chloride), we can conclude that the integrated SECM-cantilever/Echemcell provides a completely equivalent setup in terms of potentiostatic control to the one provided by macroscopic electrodes commonly found in fluid cells for electrochemistry by AFM.

### 3.4. SECM-Measurements with the Integrated Electrochemical Cell

**Figure 4:**
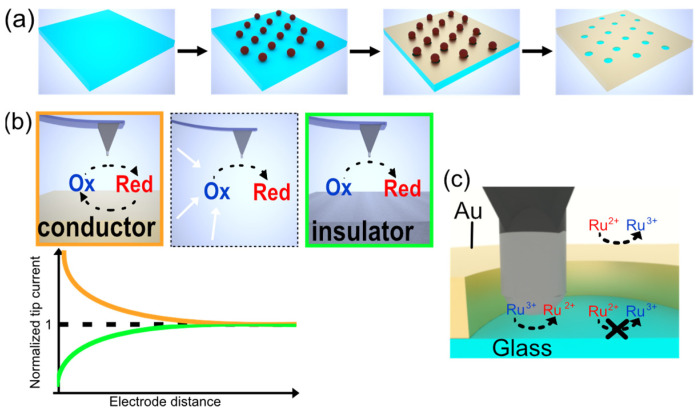
(**a**) Schematic production of gold nano-meshes used as a test sample for AFM-SECM measurements. (**b**) Working principle of SECM feedback mode under bi-potentiostatic control. Dashed lines indicate electron transfer reactions on the electrode, while white arrows indicate diffusion of redox species. The schematic graph of the corresponding normalized tip current for positive (orange) and negative (green) feedback demonstrates the dependency on tip-sample distance. The dashed line marks the diffusion-limited bulk current in the bulk. (**c**) Schematic representation of SECM feedback and electron transfer reaction on nano-mesh electrodes for the SECM experiments conducted.

The most important corroboration for the integrated SECM-cantilever/Echemcell is its performance for imaging in SECM-AFM applications. As a test substrate for SECM imaging, we did choose so-called gold nano-mesh electrodes. These electrodes have been used already in the past as model systems to determine the resolution achievable by the SECM-cantilevers [12,27]. The nano-meshes were prepared by means of colloidal lithography [46,47]. Figure 4a summarizes in a schematic manner the preparation of such nano-meshes. Briefly, a densely packed layer of colloidal polystyrene particles is transferred to a functionalized glass substrate. The particles are etched by exposure to oxygen plasma and a thin layer of gold is evaporated onto the substrate. After the mechanical removal of the polystyrene particles, a nano-mesh of gold with defined hole sizes on an insulator surface is the result. The detailed procedure utilized here has been described elsewhere more in detail [47]. These nano-meshes are excellent test samples for SECM due to their defined structure with conducting gold and insulating holes wielding a glass surface [12]. Here, we did prepare Au nano-meshes with a whole diameter of 870 nm and a center of hole to center of hole separation of 1040 nm.

During SECM measurements, various feedback modes are possible [58]. Here, the two working electrodes, i.e., the Au-nano-mesh and the SECM tip, are independently controlled by a bi-potentiostat. The SECM measurements were performed in a 5 mM solution of hexaammineruthenium(III) chloride in dual-pass mode. In the first pass, the surface topography has been obtained by PeakForceTapping in liquid. For the second scan, a constant lift-height of 40 nm has been maintained and the current of the SECM-signal in function of the lateral position has been acquired. For the measurements with the standard electrochemical cell, a negative potential of φ*_Tip_* = −0.50 V vs. SCE was applied to the SECM-tip, while maintaining a constant potential at the gold-nano-mesh electrode with φ*_Mesh_* = 0.20 V vs. SCE. Consequently, the ruthenium is reduced at the SECM tip and re-oxidized at the nano-mesh electrode.

Figure 4b illustrates the difference in the resulting currents detected when the tip is situated over the nano-mesh electrode or the insulator surface. The reduction of oxidized species occurs at the electrochemical active SECM tip when the negative potential φ*_Tip_* is applied. The current will depend on the concentration of Ru^3+^-ions. If this reduction takes place over the Au-electrode of the nano-mesh the local concentration will be higher than in the bulk due to the additional regenerated former Ru^2+^-ions. The oxidation and regeneration of Ru^2+^- to Ru^3+^-ions take place at the Au-part of the nano-mesh when the potential φ*_mesh_* is applied. However, in the insulator parts of the nano-mesh, no regeneration takes place and the Ru^3+^-ion-concentration is reduced. These two processes are also often referred to as positive and negative feedback, respectively, in SECM [58,62]. It should be noted that both processes are amplified in effect with reduced distance to the solid/liquid interface (cf. lower graph of Figure 4b). For negative feedback, the diffusion of Ru^3+^-ions is reduced compared to the bulk. For positive feedback, the regenerated concentration is highest near the electrode at φ*_mesh_*. Figure 4c summarizes the expected currents for the experiments. For the Au-part of the nano-mesh, a positive feedback signal and thus a high current is expected as schematically illustrated. By contrast, in the holes of the mesh with the glass surface, negative feedback takes place, and a reduced current should be detected. 

**Figure 5:**
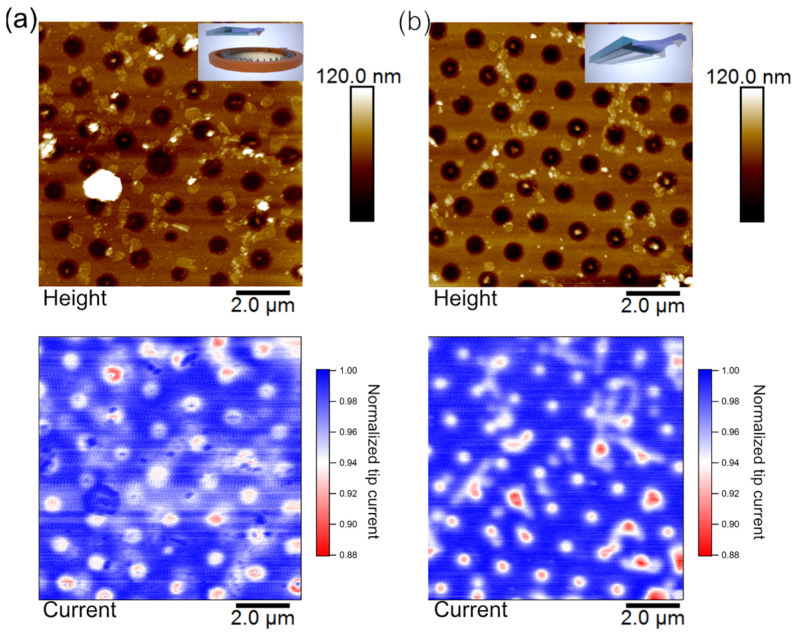
(**a**) AFM-SECM images of simultaneously acquired topography and tip current at an electrode distance of 40 nm using the ‘standard’ AFM-SECM setup with macroscopic electrodes. (**b**) AFM-SECM images acquired with the integrated SECM-cantilever/Echemcell. The current signal for SECM-current images was normalized line by line to current on gold for better visibility.

Figure 5a,b show the results of AFM-SECM measurements conducted in PeakForceTapping mode: one time in a conventional electrochemical cell with macroscopic reference and counter electrodes (cf. Figure 5a) and one time with only the integrated SECM-cantilever/Echemcell (cf. Figure 5b), respectively. In both cases, the gold nano-mesh acted as a second working electrode, leading to a regeneration of oxidized species near its surface. The height images (top graphs) show in both cases the typical features of the nano-mesh electrode. The total height was 50 nm for the Au-layer and a diameter of 870 nm for the holes in the mesh. The slight difference in the resolution between the two topographic images is attributed to the wear of the tip as Figure 5b has been acquired directly before Figure 5a by means of the same SECM cantilever.

The current images (cf. bottom of Figure 5a and 5b, respectively) show nearly exactly the same characteristics in terms of current and lateral resolution. As expected from Figure 4c, the current is reduced in the areas of holes. For both images, the detected current has been line fitted to compensate for drift effects. Instrumental drift does lead in particular to uncertainty in terms of the separation between probe and sample. The latter has a large effect on the detected currents [4]. PeakForce Tapping mode leads to a significant reduction of this effect [12,63]. Moreover, the dimension of the SECM probe is of critical importance for achievable resolution [4]. Taking into account the tip-wear during the previous imaging, no difference between the two images can be detected, illustrating the comparable performance of a classical electrochemical cell and the integrated SECM-cantilever/Echemcell. From the current image, a lateral resolution better than 250 nm can be clearly derived. This value is in line with the one reported previously for the same type of SECM cantilevers [12].

## 4. Discussion

The advent of batch fabrication and commercially available cantilevers with reproducible characteristics for SECM [12,20,32] has led to an increased interest in applying AFM-based SECM in various fields, such as battery research [17,18,19], corrosion [15], catalysis [16] or biology [5]. While the batch processing as well as the reproducible production of SECM tips were in the focus for technical developments in AFM-based SECM in recent years, we believe that it is now time to focus on the electrochemical cell. The design idea of the here-presented integrated SECM-cantilever/Echemcell is similar to recently developed electrochemical cells for in situ and in operando transmission electron microscopy (TEM) [30,55,56,64,65]. Another field where miniaturization of the electrochemical cell has taken place is the technique of scanning electrochemical cell microscopy. However, for this technique, the configuration of the electrodes is not comparable to the here presented setup. The integration of the complete electrochemical cell onto the exchangeable parts, namely the cantilever (working electrode) and its glass packaging (counter and reference electrode), will provide several advantages. First, the reference electrode is placed near the working electrode(s). For the extremely small SECM tips the ohmic drop is not highly relevant [58]. However, if the sample working electrode has only an active area of a few mm^2^, then the integrated SECM-cantilever/Echemcell might still be used as a counter electrode, which would be placed at the shortest possible distance for the second working electrode, thereby reducing ohmic drop effects. Second, the reference electrode would be placed very near to both working electrodes and be practically equidistant from both. Third, one of the biggest advantages is the resulting miniaturization of the electrochemical cell by the integrated SECM-cantilever/Echemcell. Thus, measurements in drop cells or small volume cells as favored in some biological applications (e.g., protein adsorption) become possible. A further advantage would be that the SECM working electrode can be tested with very moderate experimental effort by just immersing it in a vessel containing a solution with a suitable redox-couple and performing cyclic voltammetry. Moreover, the integrated SECM-cantilever/Echemcell would prevent the ‘running dry’ of reference electrodes, which is a common source of errors in fluid cells with macroscopic electrodes due to the resulting overpotentials that destroy often the SECM-cantilever. However, the resulting miniaturization of the whole liquid cell might lead to an overall reduction of thermal drifts, which represents a major limitation for the performance of SECM [4].

The implementation of the SECM-cantilever/Echemcell is based on pastes that have been developed for the screen printing of electrodes on glass substrates. Screen-printed electrodes have by now a long history in electrochemistry and their preparation has been optimized in recent years [66,67]. Hence, the here-used preparation process by painting the electrodes with a brush on the glass packaging can be certainly optimized. It will lead to more homogenous and thinner electrodes that can be cured in a more reliable manner. Generally, the preparation of the two additional electrodes by sieve printing could be easily integrated into the workflows of preparing SECM-cantilevers. As a result, the homogeneity, form, and area of the reference and working electrode would be better defined. If the transfer of the pastes to the glass packaging is performed before the assembly of packaging and cantilever, higher curing temperatures are achievable, thus allowing for increased chemical resistance and a wider choice of pastes for the electrodes. Nevertheless, using the here-presented preparation, resistance to chemicals should be already good enough even for solution environments common for SECM studies in battery materials [68,69,70]. Here, curing took place at temperatures below 100 °C. Screen printed Ag/AgCl electrodes are also used for various analytical and monitoring applications [71,72]. Hence, a high degree of reproducibility is ensured, and a wealth of experience in terms of the stability of such electrodes under different conditions exist. In particular, the quality of the reference electrodes is most likely sufficient for applications in AFM-based SECM. Future improvements of the design could be further miniaturization by preparing the electrodes directly on the cantilever chip instead of the glass carrier.

## Data Availability

Data is available from the corresponding author upon request.
